# Genome Modification Technologies and Their Applications in Avian Species

**DOI:** 10.3390/ijms18112245

**Published:** 2017-10-26

**Authors:** Hong Jo Lee, Young Min Kim, Tamao Ono, Jae Yong Han

**Affiliations:** 1 Department of Agricultural Biotechnology, College of Agriculture and Life Sciences, and Research Institute of Agriculture and Life Sciences, Seoul National University, Seoul 08826, Korea; cszjjang@naver.com (H.J.L.); ypoc01@snu.ac.kr (Y.M.K.); 2 Faculty of Agriculture, Shinshu University, 8304 Minamiminowa, Kamiina, Nagano 399-4598, Japan; tamaoon@shinshu-u.ac.jp; 3 Institute for Biomedical Sciences, Shinshu University, 8304 Minamiminowa, Kamiina, Nagano 399-4598, Japan

**Keywords:** genome modification, avian, transgenic technology, programmable genome editing, CRISPR/Cas9, primordial germ cells

## Abstract

The rapid development of genome modification technology has provided many great benefits in diverse areas of research and industry. Genome modification technologies have also been actively used in a variety of research areas and fields of industry in avian species. Transgenic technologies such as lentiviral systems and *piggyBac* transposition have been used to produce transgenic birds for diverse purposes. In recent years, newly developed programmable genome editing tools such as transcription activator-like effector nuclease (TALEN) and clustered regularly interspaced short palindromic repeats (CRISPR)/CRISPR-associated protein 9 (CRISPR/Cas9) have also been successfully adopted in avian systems with primordial germ cell (PGC)-mediated genome modification. These genome modification technologies are expected to be applied to practical uses beyond system development itself. The technologies could be used to enhance economic traits in poultry such as acquiring a disease resistance or producing functional proteins in eggs. Furthermore, novel avian models of human diseases or embryonic development could also be established for research purposes. In this review, we discuss diverse genome modification technologies used in avian species, and future applications of avian biotechnology.

## 1. Introduction

Genome modification in living organisms provides several advantages for research and industrial purposes. Transgenic technology, including lentiviral systems [1] and *piggyBac* transposition [2], has contributed to the development of transgenic animals for exogenous gene expression. Additionally, the development of revolutionary genome editing tools such as zinc finger nuclease (ZFN) [3], transcription activator-like effector nuclease (TALEN) [4], and clustered regularly interspaced short palindromic repeats (CRISPR)/CRISPR-associated protein 9 (CRISPR/Cas9) [5] have made it easy to precisely edit genome information. These tools are expected to contribute to the establishment of customized organisms for specific purposes through programmable genome editing. Applications of the genome editing technologies enable the exploration of unknown gene functions through targeted gene disruption, or they can be used for the development of therapies for genetic disorders through genome replacement. In agriculture, genome editing has been adopted as “precision breeding” using these technologies to recreate biological traits such as growth rate, disease resistance, and the production of bio-functional protein [6].

The development of new genome modification technologies is also creating a surge in the field of avian biotechnology. Due to their enormous potential in diverse disciplines, adaptations of genome editing technology to the avian genome are in high demand. Production of avian lines with disease resistance or the ability to produce functional egg proteins are the most highly desired results in avian biotechnology, as well as their use as an animal model for developmental studies [7]. As a result of long-term efforts in avian biotechnology, some success in avian genome modification has been achieved, and it will be further facilitated in the near future.

In this review, we cover the historical advances in genome modification technology and its applications to biotechnology, particularly in avian biotechnology. Future strategies in avian genome modification technology are also discussed.

## 2. Development of Transgenic Technologies and Application for Avian Genome Modification

Developing designed living organisms has many benefits for both research and industry. Genetically modified organisms, in particular, have tremendous benefits for the field of biological research. Transgenic technology has developed rapidly since the creation of the first genome-modified mouse by DNA virus injection into an early-stage mouse embryo was reported in 1974 [8]. In 1981, Gordon et al. produced the first transgenic mouse with germline transmission by injecting purified DNA into the pronucleus of a mouse embryo [9]. Although this method has limitations in germline transmission efficiency and random integration, it has been widely used to produce transgenic mice.

In addition to pronuclear injection, viral vector systems have also largely contributed to the development of transgenic animals. Viruses transport their viral genome into cells, and exogenous DNA is easily transduced into host genomes of infected cells using a modified viral genome. The development of several types of viral vector systems using retroviruses, adenoviruses (ADV), herpes simplex viruses (HSV), and adeno-associated viruses (AAV) facilitated the use of viral vectors as transgene over-expression systems for diverse purposes, including gene therapy as well as transgenesis [10].

The first report of viral DNA transduction was published in 1976, when a hybrid virus containing Simian virus 40 (SV40) and lambda phage DNA was transduced into cultured monkey cells [11]. After the first use of a viral vector for DNA delivery was reported, diverse viral vectors were developed for transgenic research. Notably, depending on the viral vector system, the maximum length of an exogenous DNA insert varies (8–10 kilobase pairs (kb) for retroviruses, more than 100 kb for HSV, 8 to 30 kb for ADV, and less than 5 kb for AAV), and the integration profiles are different [12]. With its relatively high packaging capacity and efficient integration profiles compared to ADV and AAV (episomal expression), viral vectors using retroviruses have been widely used in transgenic research [13]. In particular, lentiviruses, a subclass of retroviruses, have advantages for integration compared to other retroviruses. Most retroviruses can only integrate their viral genome into the genomes of dividing cells; however, lentiviruses can also integrate their viral genomes into the genomes of non-dividing cells [14]. This distinguishing characteristic of lentiviruses enables the use of this viral system for genome modification as well as exogenous gene over-expression.

As a result of a highly efficient genome modification capacity, these viral systems have also been used for avian transgenesis. After the first studies of transgenic animal production by retroviral infection in Eyal giladi and Kochav (EG&K) stage X chicken embryos, diverse transgenic birds have been successfully established for a variety of purposes, including functional protein expression and the development of model animals [15,16,17,18,19,20,21]. Specifically, mass production of functional proteins using retroviral systems in serum and egg whites of transgenic chickens suggests its practical use as a bioreactor system, although the silencing of transgenes was observed in the subsequent generation (~5.6 mg/mL) [22]. Moreover, oviduct-specific expression of therapeutic proteins in transgenic chickens was successfully achieved using a lentiviral system by delivering a transgene containing ovalbumin regulatory sequences [16]. The viral system has been shown to produce other transgenic birds in addition to chickens, including quail and zebra finches, demonstrating its versatility in the transgenesis of avian species [17,23,24].

Despite the high efficiency of genome modification in living organisms, viral infection systems have fundamental limitations, such as their use in food proteins for human consumption and random exogenous gene integration in host cells causing unexpected results with low germline transmission efficiency and silencing effects in transgenic animals [22,25,26]. To overcome these obstacles, a transgenic technology using transposons was developed. Transposons are mobile DNA elements that take up a large portion of the genome. These mobile elements move between genomes, and in certain cases, have important roles in genome function and evolution through the insertion of certain chromosomal regions. The characteristics of these elements make them useful tools for DNA integration into host genomes as well as in gene trap studies [27].

One of these elements, the *piggyBac* transposon isolated from the cabbage looper moth (*Trichoplusia ni*), has been used for genome modification in living organisms [28]. It recognizes TTAA sequences in the genome and inserts into such sites [29], therefore, diverse integration of transposons is possible. Transposon-mediated DNA introduction is very effective in mice and humans, and reports suggest that the germline transmission efficiency of transposons is higher than in viral systems [2,30]. Another transposon actively used for genome modification is *Tol2*, isolated from medaka fish (*Oryzias latipes*) [31]. The *Tol2* transposons also have “cut and paste” mechanisms similar to other transposons, and they have been successfully used in diverse vertebrate cells from zebrafish, frogs, mice, and humans [32]. In addition, a synthetic DNA transposon, the Sleeping Beauty transposon, has been used as an exogenous gene delivery system. It was artificially synthesized from the genomes of salmonid fish [33]. Similar to other transposon systems, it can also recognize specific DNA sequences and integrate into the host genome.

Efficient transgenic technology has been successfully adapted for the production of transgenic chickens with PGC transplantation or direct injection in vivo [34,35,36,37,38,39]. Our previous studies reported that *piggyBac* transposition was effective in producing transgenic chickens mediated by transplantation of genome-modified PGCs (over 90% germline transmission efficiency) [38,39], and the system was used to develop a chicken model combined with site-specific recombination technology [36]. Not only *piggyBac* transposon-, but also *Tol2* transposon-mediated transgenic chickens have been reported. The reports showed that *Tol2* transposons integrated into the genomes of chicken cell lines, including the PGCs, in an efficient manner (45.2%) [37], and even direct in vivo injection of *Tol2* transposons and transposase plasmids into the bloodstream of chicken embryos was successfully used to produce transgenic chickens, despite low germline transmission efficiency (<2%) [35,40].

## 3. Homologous Recombination Technology for Gene Targeting

Although transgenic technology provides opportunities for developing useful model organisms in a highly efficient manner, this technology also has fundamental limitations due to random integration. With precise genome editing technology under high demand, the first homologous recombination technology adapted to mouse embryonic stem cells (ESCs) was reported [41]. Homologous recombination is one of the natural DNA repair mechanisms in living organisms, along with non-homologous end joining (NHEJ), which occurs during meiosis, resulting in genetic diversity between parents and their offspring. To adopt this phenomenon to gene targeting technology, donor plasmids should contain DNA homology sequences for targeted loci with proper selective markers. DNA homology in the donor plasmid can induce targeted replacement between the host genome and donor plasmid; therefore, the length of a DNA homology arm is critical for recombination efficiency, although the efficiency could vary depending on the targeted locus [42]. Although the efficiency of homologous recombination technology was extremely low (0.001%) compared to transgenic technology for genome modification, attempts to enhance homologous recombination events by transient alteration of gene expression relating to DNA repair mechanism, specifically RAD52, have been performed [43]. Furthermore, the development of precise genome editing mediated by homologous recombination provides tremendous benefits for studying specific gene functions in living organisms.

The first knockout chicken was developed using this classical gene targeting technology [44]. The report showed that the joining (J) gene segment of the chicken immunoglobulin (Ig) heavy chain gene was targeted by homologous recombination in the genome of PGCs, and knockout chickens were produced by PGC transplantation. The efficiency of gene targeting of the Ig gene of PGCs was about one targeted clone per 10^7^ cells (0.00001%), and germline transmission of knockout clones was extremely low (0.005–0.2%). The results indicated that the combined gene targeting technologies of homologous recombination and chicken PGC culture systems was a promising method for producing gene-targeted chickens, but required improvement in terms of efficiency for further research.

## 4. Site-Specific Recombination

With homologous recombination technology, site-specific recombination using recombinases has contributed greatly to the field of genome editing technology. By inserting recombinase recognition target sites into donor plasmids, targeted regions were modified with recombinase treatment. The technology is called “conditional regulation”, which is a useful tool for studying genes that are critical for cell survival [45].

The most well-known site-specific recombinase is the Cre-loxP system. Cre recombinase, derived from the P1 bacteriophage [46], recognizes loxP sequences and modifies the sequences floxed by loxP sequences. When identical loxP sequences lay in the same orientation in the genome, Cre cleaves the floxed sequences (excision). When the same loxP sequences lay in different orientations, the floxed sequences are inverted (inversion). When two different loxP sequences lay in the same orientation, the floxed sequences are replaced when donor plasmids containing the same two different loxP sequences are used (recombinase-mediated gene cassette exchange; RMCE) [47]. Flipase/Flipase recognition target (Flp/*FRT*) system is an additional site-specific recombination technology. The system is derived from yeast (Saccharomyces cerevisiae) and can lead to genetic recombination [48]. The recombination depends on the FRT orientation, and the mechanism is similar to the Cre-loxP system. Two site-specific recombination systems have been used in diverse organisms, including mice and cows [49,50].

In avian species, site-specific recombination mediated by Cre-loxP was used to express monoclonal antibodies driven by the ovalbumin promoter in primary oviduct cells [51], and recently a transgenic chicken expressing Cre recombinase was produced for targeted excision of the loxP-floxed genome [52]. In both cases, Cre-loxP recombination was successfully adapted for genome rearrangement (excision), indicating its possible application for obtaining desired genotypes in avian species. Our group also demonstrated RMCE in transgenic-derived cells using the Flp/*FRT* recombination system [36]. The successful genome replacement on predetermined loci of a transgenic chicken genome suggests that it could be used to induce the expression of exogenous proteins at predictable levels in transgenic chicken lines.

Another well-known site-specific recombinase is PhiC31 integrase. It is derived from bacteria, and recognizes and modifies PhiC31-specific attP and attB sites. The recombinase recognizes, rotates, and pastes double strands of the floxed genome, and the attP and attB sites are converted to attL and attR that do not react with the PhiC31 integrase [53]. Therefore, unlike the Cre-loxP and Flp/*FRT* recombination systems, the recombination is irreversible. This recombination system is used as a cloning tool, called “gateway cloning” [54]. Site-specific recombination technology using PhiC31 integrase has also been used for transgene integration in avian species. Humanized antibody expression was induced by PhiC31 integrase with homologous recombination in the chicken B cell line, DT40 [55], and Cre-expressing transgenic chicken was mediated by adapting the technology to chicken PGCs [52]. The irreversible recombination of this technology may aid in establishing desired clones without further genome alteration.

## 5. Programmable Genome Editing Using Endonucleases

In biology, the emergence of programmable genome editing technology is considered the most remarkable event of the 21st century. Precise recognition and efficient cleavage of double strand DNA using programmable genome editing tools opened a new era of biotechnology. The tools not only induce loss-of-function of the gene, but also enhance homologous recombination by double-strand DNA breakage. Furthermore, the synthesis of these tools is becoming increasingly convenient, as thousands of customized vectors are now available.

The first generation of programmable genome editing tools was ZFN. ZFN is composed of zinc finger motifs that can bind to DNA nucleotides, and the FokI endonuclease, found in *Flavobacterium okeanokoites*, is involved in the double-strand breaks [3]. Zinc finger motifs can bind three nucleotides depending on amino acid composition, and pairs of ZFN motifs are more effective in DNA cleavage [56]. Furthermore, combinations of several ZFNs enable recognition and mutation of specific genomic loci [57]. ZFN has been widely used in genome editing in living organisms, including insects, plants, and even human cells [58].

The second generation of programmable genome editing tools is TALEN. TALEN is composed of a TAL effector that is derived from pathogenic bacteria, *Xanthomonas*, and the same FokI endonuclease used in ZFN [4]. TALEN also recognizes and induces double-strand breaks of targeted nucleotides, and the TAL effector has a critical role in recognizing nucleotides. The DNA binding domain of the TAL effector contains 33–34 conserved amino acid sequences with a variable region at amino acid positions 12 and 13, called the repeat variable di-residue (RVD). Depending on the amino acids of the RVD, a TAL effector recognizes different nucleotides (HD binds to C, NG binds to T, NI binds to A, and NN binds to A or G) [4]. Unlike ZFN, each TAL effector containing one repeat domain can bind to one nucleotide, thus it has an advantage over ZFN in target site design [59]. Because TAL effectors can combine with activator domains, such as transcriptional activators and endonucleases, TALEN has been used widely for diverse purposes in diverse organisms [60]. With the development of the TALEN assembly kit using golden gate shuffling, customized TALEN is now easily synthesized [61,62,63,64].

We successfully used TALEN to produce chickens with a mutant ovalbumin gene. As a programmable genome-editing tool, TALEN breaks double-stranded genomic DNA and induces NHEJ [65]. NHEJ occurs more frequently than homology directed repair in nature; therefore, TALEN exhibits a higher efficiency in introducing indel mutations than homologous recombination-mediated genome editing. Our chicken ovalbumin gene knockout using TALEN was highly effective (~60% in DF-1 fibroblasts and ~36.7% in chicken PGCs), and the germline transmission efficiency also improved drastically (~53.2% germline transmission and ~10.4% genome-edited chicken production) compared to classical gene targeting technology using homologous recombination (0.005–0.2% efficiency) without genomic integration of exogenous DNA [44,66]. More recently, TALEN-mediated gene targeting was applied to chicken *DDX4* gene disruption. The study showed that the *DDX4* gene was successfully replaced by TALEN-mediated homologous recombination in PGCs (8.1%), and genome-edited chickens were produced by transplanting PGCs (~6%). Disruption of the *DDX4* gene caused loss of PGCs in female chickens, but heterozygous male chickens remained fertile [67]. The enhanced genome editing efficiency compared with previous research suggests that the induction of double-strand breaks by endonucleases is an efficient tool for precise genome editing in avian species [44,67].

The third generation of programmable genome editing tools is CRISPR/Cas9. CRISPR and Cas9 are components of prokaryotic DNA that have important roles in the bacterial immune system. In nature, bacteria have clustered repeats called CRISPRs that are identical to viral genomes. When a virus invades a cell, a CRISPR binds to viral RNA and disrupts it with the Cas9 protein [68]. The CRISPR/Cas9 system also recognizes and induces double-strand breaks in targeted DNA sequences, similarly to ZFN and TALEN. To recognize and disrupt targeted nucleotides, the CRISPR/Cas9 system requires the Cas protein, CRISPR RNA (crRNA), and trans-activating CRISPR RNA (tracrRNA) [5]. Unlike ZFN and TALEN, CRISPR/Cas9 does not require paired units to induce double strand breaks. Moreover, as synthesis of crRNA and tracrRNA is relatively simple, thousands of customized CRISPR/Cas9 systems for targeting genes have been constructed. Owing to the convenience of CRISPR/Cas9 vector construction, this programmable genome editing tool has been used in most living organisms [69].

As the most versatile tool for genome editing, the CRISPR/Cas9 system has also been successfully adopted in many avian species, including chicken and quail. CRISPR/Cas9 has been successfully used to modify genes with high efficiency in avian somatic cells, chicken ESCs, and spermatogonial stem cells (SSCs) [70,71,72,73,74]. Furthermore, introduction of CRISPR vectors into PGCs succeeded in producing genome edited chickens with indel mutations in the ovomucoid gene and replaced gene cassettes in the chicken immunoglobulin gene [75,76]. This study showed that targeted gene knockout mediated by CRISPR/Cas9 was very effective in chicken PGCs (~100%), and germline transmission was also relatively higher (~58%) compared with TALEN [66,76]. Germline transmission was more efficient with CRISPR-mediated knock-in (~48%) than TALEN-mediated knock-in [67,75]. These results indicate that this revolutionary tool is the most effective programmable genome editing technology for avian species to date, see Table 1.

In addition to the development of precise and efficient genome editing tools such as CRISPR/Cas9, new technologies for enhancing genome-editing capacity have been developed. CRISPR from *Prevotella* and *Francisella 1* (Cpf1) is an example. It is also derived from the bacterial immune system, and has similar effects to the CRISPR/Cas9 system. The Cpf1 protein, which has RuvC domains that are responsible for cleaving DNA strands, works with Cpf1-specific single CRISPR guide RNA. In contrast to CRISPR/Cas9, protospacer adjacent motif (PAM) sequences of Cpf1 guide RNAs (TTN or TTTN depending on the bacterial strain) are different to CRISPR/Cas9 (NGG, NNAGAAW or NNNNGATT depending on the bacterial strain) [77,78], and the cleavage site is separate from the PAM sequence [78]. The cleavage pattern suggests that CRISPR/Cpf1 can be used to target different sites simultaneously using several gRNAs that cannot be modified by CRISPR/Cas9, and genome editing mediated by homologous recombination may be enhanced compared to the CRISPR/Cas9 system. Moreover, several studies using genome-wide analysis such as Digenome-seq have reported that CRISPR/Cpf1 has lower off-target effects—which is the main problem for genome editing in all species—than CRISPR/Cas9, thus it is considered more suitable for gene correction [79,80].

In addition to Cpf1, applications combined with CRISPR/Cas9 have also been reported. These studies introduced CRISPR-cytidine deaminase complexes, which convert cytidine to other nucleotides without indel mutations on targeted loci, called “base editing” [81,82]. The base editing system could be applied to gene correction without homologous recombination technology or indel mutations.

## 6. Future Directions in Avian Biotechnology

Over the past few decades, the development of genome editing technologies has brought about many changes in the field of biology. In avian species, genome-editing technology has enabled precise and efficient modification of the avian genome, and now, it is expected to maximize the value of the avian system for industrial and research purposes.

One of the most highly anticipated goals of avian technology is to establish valuable chicken lines with high growth and egg production rates through genome editing technology, called “precision breeding” [6]. Because the chicken is a highly productive protein resource for humans, enhancing its economic traits through genome editing will be helpful in providing a low-cost food source. Not only could the amount of protein be increased, but the quality of chicken protein could also be improved through genome editing. By modifying egg protein genes such as ovalbumin and ovotransferrin, as well as other egg proteins, diverse properties such as nutrient levels, allergenicity, or the production of bio-functional materials in eggs could be regulated. Furthermore, mass production of valuable functional proteins would be possible through precise gene insertion into endogenous egg protein coding sequences with genome editing technology [7]. These new breeding approaches could yield great benefits to human society.

**Figure 1:**
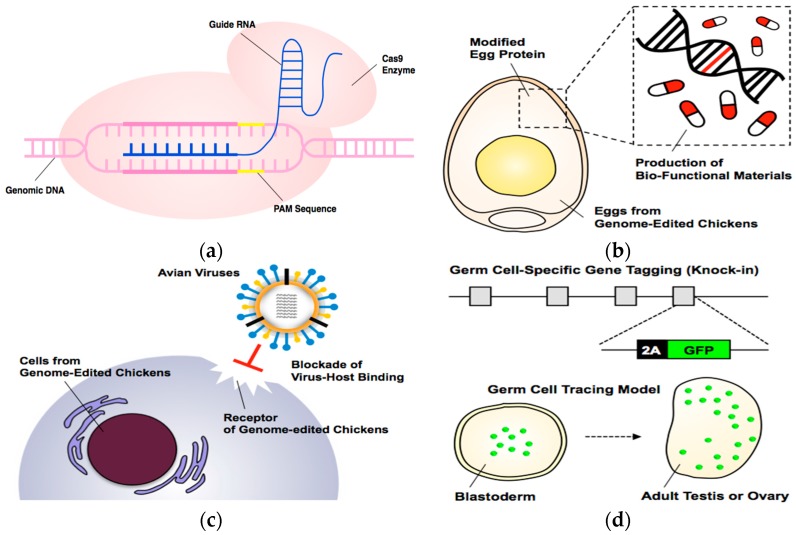
(**a**) CRISPR/Cas9 system for programmable genome editing. Guide RNA specifically binds to genomic DNA, and then Cas9 enzyme breaks double strand of DNA adjacent to protospacer adjacent motif (PAM) sequence; (**b**) Genome modification of egg protein coding genes for production of bio-functional materials; (**c**) Precise genome modification of host receptor for preventing viral infections, T arrow indicates blockade of virus-host binding; (**d**) CRISPR/Cas9-mediated knock-in of fluorescent protein gene to germ cell specific genes (tagging) for establishment of germ cell tracing model following embryogenesis (dashed arrow).

Another expected outcome for avian genome editing technology is the development of disease resistant avian lines. Pandemic diseases such as avian influenza (AI) and avian leukosis virus (ALV) have caused enormous damage to the poultry industry and to the general population. Previously reported research has suggested that overexpression of small hairpin RNA specifically regulating viral RNA polymerase expression may prevent AI transmission [83], suggesting that genome editing is the one of most promising strategies for disease resistance in avian species. Alternatively, genome editing of host receptors could be applied for disease resistance. Fortunately, the host receptors of AI and ALV have been well described, and our previous results suggest genome mutations on host receptor alter disease resistant in chicken cell line [84,85,86,87,88,89,90]. Furthermore, the strategy was proven in human immunodeficiency virus (HIV) by genetic mutations in the CCR5 host receptor gene [91]. With genome editing of these receptors, modified organisms could be easily evaluated in vitro and in vivo, and such studies could contribute to the establishment of disease-resistant chickens (Figure 1).

Despite the rapid development of genome editing technology, there is still much room for improvement. Germline transmission in avian species largely depends on PGC transplantation methods, and long-term in vitro culture of germline-competent cells is a prerequisite for precise genome editing in avian species [92]. Although there have been many successful reports on long-term culturing of PGCs following genome editing, the methods are time-consuming and require great skill [93,94]. To establish highly germline-competent cell lines, in-depth studies on the cells, involving signaling pathways, gene expression, and the development of new in vitro culture systems, should be performed. Alternatively, direct gene injection into the embryonic bloodstream or testis is a potential method for inducing germline transmission [40]. Transplantation of genome-edited testicular cells or SSCs into adult chicken testis may reduce the length of time needed to establish genome-edited chickens [95,96]. Furthermore, a recent study reporting sperm transfection-assisted gene editing (STAGE) suggested the utilization of bird sperm as a mediator for genome modification in avian species [97].

## 7. Conclusions

The advent of genome editing technology heralds a new era in avian biotechnology. Because avian species have a great deal of merit in diverse fields due to their unique biological characteristics, the variety of research involving genome editing in avian species using these genome editing technologies is expected to increase. The development of avian biotechnology will contribute to the development of diverse research disciplines and fields of industry benefitting human society.

## Figures and Tables

**Table 1:** Comparison of programmable genome editing technologies adopted in avian species.

Methods	Efficiency of Genome Editing in Chicken PGCs	Efficiency of Germline Transmission (Genome-Edited Chickens)	References
TALEN	33.3%	22.3–53.2% (0.0–10.4%)	[66]
CRISPR/Cas9	0–100%	67–79% (48–58%)	[76]
Homologous recombination	0.00001%	0.005–0.2% (NA ^1^)	[44]
TALEN + homologous recombination	8.1%	NA ^1^ (0–6%)	[67]
CRISPR/Cas9 + homologous recombination	20–33%	0–96% (0–48%)	[75]

^1^ Not available.

## Abbreviations

TALEN	Transcription activator like effector nuclease
CRISPR/Cas9	Clustered regularly interspaced short palindromic repeats (CRISPR)-associated protein 9
ADV	Adenoviruses
HSV	Herpes simplex viruses
AAV	Adeno-associated viruses
SV40	Simian virus 40
kb	Kilobase pairs
EG&K	Eyal giladi and Kochav
PGC	Primordial germ cell
ZFN	Zinc finger nuclease
ESC	Embryonic stem cell
NHEJ	Non-homologous end joining
RMCE	Recombinase mediated gene cassette exchange
Flp/*FRT*	Flipase/Flipase recognition target
RVD	Repeat Variable Di-residue
crRNA	CRISPR RNA
tracrRNA	Trans-activating CRISPR RNA
PAM	Protospacer adjacent motif
AI	Avian influenza
ALV	Avian leukosis virus
Cpf1	CRISPR from *Prevotella* and *Francisella 1*
GUIDE-seq	Genome-wide, unbiased identification of double strand breaks enabled by sequencing
SSCs	Spermatogonial stem cells
HIV	Human immunodeficiency virus
STAGE	Sperm transfection-assisted gene editing
